# Multisession transcranial direct current stimulation and aerobic exercise synergistically improve food craving symptoms, impulsivity, and cognitive flexibility in women with overweight and obesity: a randomized controlled trial

**DOI:** 10.1186/s12966-025-01773-0

**Published:** 2025-06-02

**Authors:** Sahar Malek Khataei, Ehsan Amiri, Daniel Gomes da Silva Machado

**Affiliations:** 1 Exercise Metabolism and Performance Lab (EMPL), Department of Exercise Physiology, Faculty of Sport Sciences, Razi University, Kermanshah, Iran; 2 Research Group in Neuroscience of Human Movement (NeuroMove), Department of Physical Education, Federal University of Rio Grande do Norte, Natal, RN Brazil; 3 Human Performance Research Center (HPRC), Department of Sport Science, University of Konstanz, University Street 10, 78464 Konstanz, Konstanz Germany

**Keywords:** Appetite, Non-invasive brain stimulation (NIBS), Physical activity, Risky decision-making, Cognitive function, Long-term potentiation

## Abstract

**Background:**

We explored the potential synergistic effects of combining multisession anodal transcranial direct current stimulation (a-tDCS) with chronic aerobic exercise (AE) on food cravings (FC), impulsivity (IMP), risky decision-making (RDM), and cognitive flexibility (CF) in women with overweight or obesity exhibiting food craving symptoms.

**Methods:**

Thirty-six women with overweight or obesity and symptoms of food craving (age: 26±6,4 years) were randomly allocated into three groups using permuted block randomization (*n* = 12 each): (1) a-tDCS + AE, (2) Sham + AE, and (3) Control (no intervention). During Phase 1, the a-tDCS + AE group received five consecutive sessions of a-tDCS, while the Sham + AE group received sham tDCS. In Phase 2, both the a-tDCS + AE and Sham + AE groups completed three sessions of moderate-intensity continuous aerobic exercise per week for four weeks. Outcome measures, including food cravings (FC) and cognitive flexibility (CF), were assessed at baseline, after five days of tDCS, and after four weeks of AE. Follow-up measurements for FC and CF were also conducted one month post-intervention.

**Results:**

FC was lower in the a-tDCS + AE group compared to Sham + AE and Control groups in Phase 1 (Cohen’s d = 1.4 and 1.9, respectively). In Phase 2, a-tDCS + AE and Sham + AE groups showed lower FC than the Control group (d = 3.8 and d = 2.8, respectively), and a-tDCS + AE also showed a lower FC compared to the Sham + AE group (d = 1.5). FC remained lower in the a-tDCS + AE group compared to Sham + AE and Control groups at follow-up (d = 1.7 and d = 2.4, respectively). CF was higher in the a-tDCS + AE compared to Sham + AE and Control groups (d = 2.1 and d = 1.4, respectively) and in the sham + AE (d = 1.0) compared to control in Phase 2. At follow-up, CF was higher only in the a-tDCS + AE group compared to the Control (d = 1.2). IMP scores were higher in the a-tDCS + AE group compared to the other groups in Phases 1 (d = 1.0 and d = 1.4) and 2 (d = 5.4 and d = 1.9). RDM was higher in the a-tDCS + AE compared to the Control group in phase 2 (d = 1.3).

**Conclusions:**

Multisession a-tDCS combined with four weeks of moderate AE synergistically reduces food cravings and improves related variables to a greater extent than AE alone, with sustained effects, in women with overweight or obesity and symptoms of food craving.

**Trial registration:**

This study was registered in the Iranian Registry of Clinical Trials (IRCT id: IRCT20210617051606N7; Registration date: 04.02.2023).

**Supplementary Information:**

The online version contains supplementary material available at 10.1186/s12966-025-01773-0.

## Background

Food craving is a strong desire or urge to consume specific foods [1, 2]. The role of food craving in overweight and obesity has long been a subject of debate [3]. While some researchers argue that it does not play a significant role in weight gain, others believe it has become increasingly influential, particularly in recent years, with easier access to high-calorie foods [2]. Ultimately, findings from multiple studies highlight that food craving is clinically associated with overweight and obesity, and its examination can enhance understanding of the contributing factors and inform effective weight management strategies [2–5]. Despite the seemingly simple terminology, the neural mechanisms underlying food craving and its associated effects remain complex, underscoring the need for a multidimensional management approach [6, 7]. Electrophysiological and neuroimaging studies emphasize the critical role of the prefrontal cortex (PFC), particularly the dorsolateral prefrontal cortex (DLPFC), in the regulation of food craving [1, 8–10]. The DLPFC is involved in higher-order functions such as working memory, cognitive flexibility, risk-taking, moral decision-making, self-control, internal inhibition, impulsivity, and problem-solving [10–13]. Reduced DLPFC activity has been associated with heightened food craving in individuals with obesity, whereas increased activation in this region supports greater inhibitory control and improved self-regulation of food intake [10].

Reduced activity in the DLPFC has also been linked to impulsive decision-making [14]. Defined as the tendency to act with minimal deliberation or control [15], impulsivity is associated with increased food cravings, binge eating, eating driven by external food cues, and high-calorie fast food consumption, ultimately heightening the risk of obesity [16]. Evidence also suggests that cognitive flexibility, encompassing both proactive and reactive control and regulated by the DLPFC, is frequently impaired in individuals with obesity, leading to stronger food cravings and diminished inhibitory control [17]. As a result, many interventions targeting food cravings aim to address these regulatory factors.

Regular physical activity is recognized as an effective intervention for managing overweight and obesity [18, 19]. Research indicates that exercise improves physiological and hormonal factors associated with obesity and also induces beneficial psycho-cognitive changes, reducing food craving and enhancing cognitive control over appetite [20, 21]. For example, a 15-minute brisk walk has been shown to reduce the urge for sugary snacks in overweight individuals [22] and 12 weeks of aerobic exercise have been found to decrease food craving [23]. Moreover, exercise may improve impulsivity [24], cognitive flexibility [25], and decision-making [26] by enhancing activity in the PFC and DLPFC, regions essential for regulating food craving [24–26].

At the same time, transcranial direct current stimulation (tDCS), a non-invasive brain stimulation technique that uses a weak electric current to modulate neuronal excitability, has also been used to target food craving and related neural mechanisms [10, 27]. For example, a recent meta-analysis highlights tDCS’s versatility in reducing food craving [27], suggesting that enhanced DLPFC activity through tDCS may improve intra-network connectivity, co-activate the executive control network, and induce neural plasticity [27–29], all of which are associated with reduced cravings. Moreover, DLPFC-targeted tDCS has been shown to improve impulsive behavior, risk-taking, cognitive flexibility, and inhibition, factors that play key roles in controlling food cravings [15, 30–35].

Taking this into account, incorporating a tDCS protocol into a physical activity program may produce synergistic effects, potentially resulting in greater reductions in food craving among individuals with overweight and obesity. Moreover, consecutive tDCS sessions (multisession tDCS) may generate cumulative effects that persist for weeks or even months post-intervention [36–38], accentuating its potential for long-term food craving and weight management. Though the synergistic or additive effects of tDCS and aerobic exercise have been reported in other populations for outcomes such as cognitive performance, executive function, and pain perception [39–42], to our knowledge, no study has examined their effects on food craving and related behavioral factors in individuals with overweight and obesity and food craving symptoms. Therefore, we explored the effects of five sessions of tDCS followed by four weeks of aerobic exercise on food cravings, impulsivity, risky decision-making, and cognitive flexibility in women with overweight and obesity and food craving symptoms. We hypothesized that this intervention would reduce food cravings and improve impulsivity, risky decision-making, and cognitive flexibility compared to exercise alone and a control group.

## Methods

### Trial design

The study employed a between-subjects design with parallel groups (three-arm) and a 1:1 allocation ratio. Participants were recruited via social media advertisements and face-to-face meetings at health centers in Kermanshah, Iran. They were invited to join the study without prior knowledge that food craving was the main focus, to minimize bias during the initial assessment of inclusion criteria. Interested individuals completed a three-part questionnaire covering demographics (age, height, weight, contact details), the 15-item Food Craving State Questionnaire (FCQ-S), and the 12-item Craving Visual Analog Scale (CVAS) color picture questionnaire [43]. Next, they attended a laboratory familiarization session, where they received instructions on the experimental procedures, brain stimulation, aerobic exercise, and research measures. Although participants initially self-reported their height and weight, these measurements were objectively verified by the investigator during this session to ensure compliance with the inclusion criteria. Participants also completed the Physical Activity Readiness Questionnaire and received detailed instructions regarding the exercise protocol. One week later, baseline assessments of food cravings, impulsivity, risky decision-making, and cognitive flexibility were conducted.

**Fig. 1:**
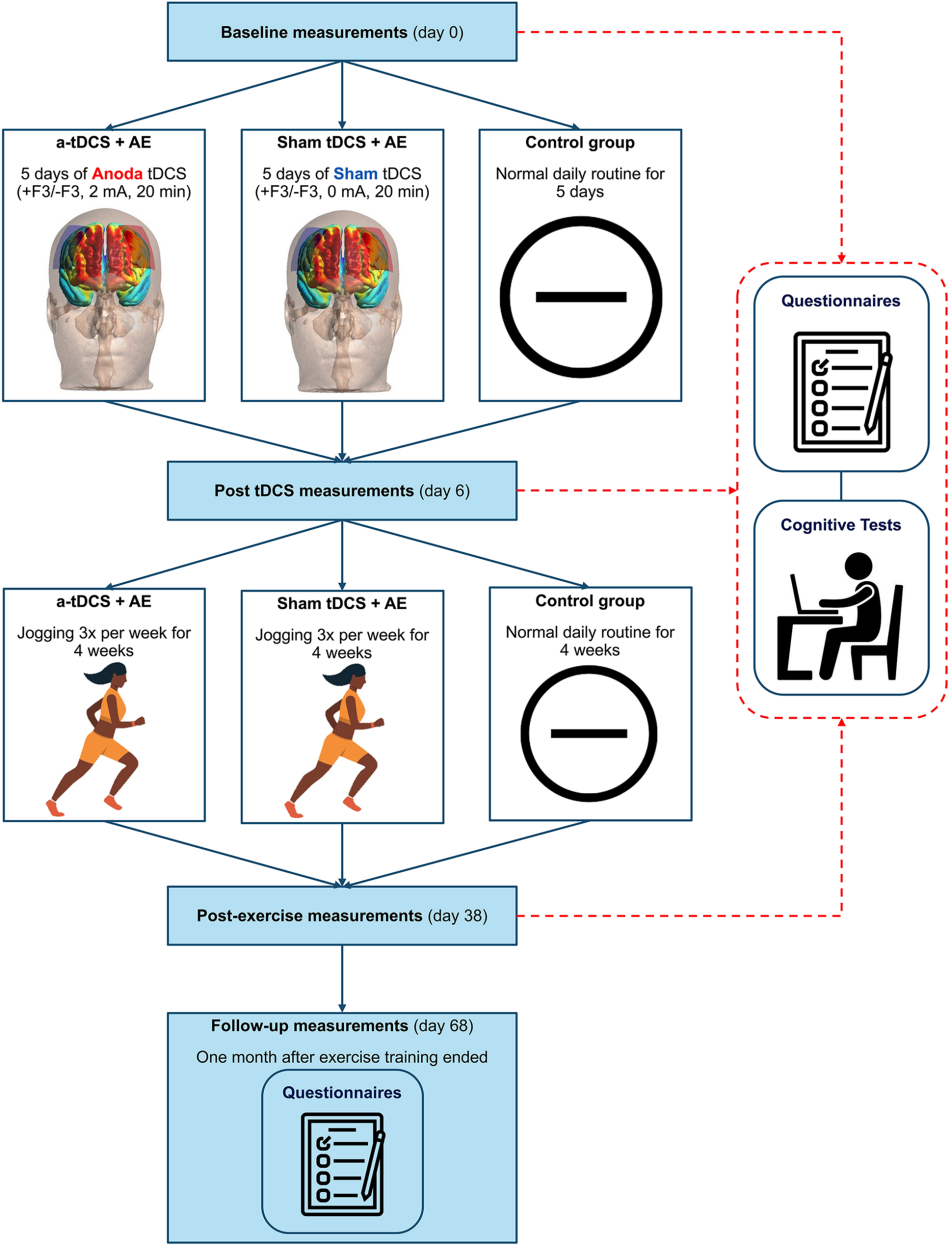
Study flow chart

Participants were then assigned identification codes and randomly allocated (via www.random.org) to one of three groups: [1] anodal tDCS + aerobic exercise (Anodal + AE); [2] sham tDCS + aerobic exercise (Sham + AE); or [3] control. The Anodal + AE and Sham + AE groups received five consecutive sessions of either anodal or sham tDCS. Food cravings, impulsivity, risky decision-making, and cognitive flexibility were reassessed 24 h after the final tDCS session. The intervention groups then completed four weeks of aerobic exercise. Forty-eight hours after the final exercise session, participants returned to the lab for the follow-up assessment under conditions similar to baseline. Four weeks after the intervention period, food cravings and cognitive flexibility were assessed again using online questionnaires due to laboratory access limitations. The control group received no interventions and maintained their usual routines throughout the study. All interventions were delivered by a clinical exercise physiologist. Both participants and outcome assessors were blinded to the stimulation condition in the experimental groups (double-blind design). The study flow is illustrated in Fig. 1.

### Participants

**Fig. 2:**
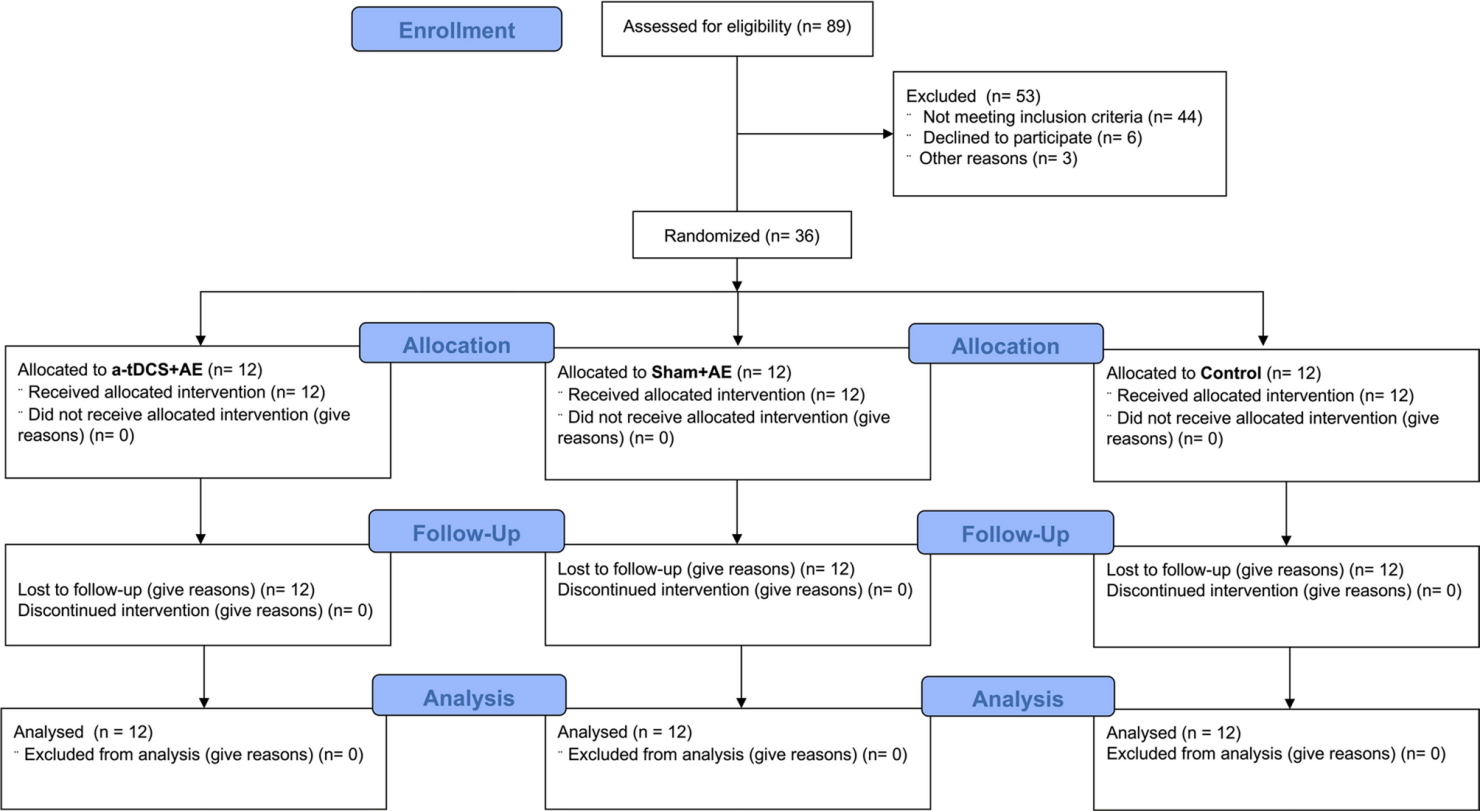
Participants’ flow during the study

Thirty-six women with overweight and obesity and food craving symptoms participated in this randomized, double-blind, sham-controlled trial. The sample size was calculated by a priori analysis in the G*Power software (version 3.1.9.2) for an ANOVA repeated measures test, with α = 0.05, power = 0.80, effect size f = 0.3 [27], three groups, four measurements, and a correlation of 0.5. Twenty-four participants were deemed sufficient, but 36 were recruited to account for potential dropouts [44]. Inclusion criteria were (1) females aged 18–50; (2) with overweight or obesity (BMI 25–34.9 kg/m²); (3) food craving symptoms [score ≥ 108 (sum of the scores of the 15-item FCQ-S and 12-item CVAS color picture questionnaire)] [36]; (4) right-handed; (5) cleared for exercise. Exclusion criteria: (1) neurological disorders (e.g., seizures, epilepsy); (2) implantable devices; (3) tobacco, alcohol, or drug use; (4) musculoskeletal disorders; (5) recent exercise or weight loss programs; and (6) menopause. The participants’ flow diagram is presented in Fig. 2. The participants’ flow diagram is depicted in Fig. 2, and their characteristics are given in Table 1. The study was approved by the Institutional Ethics Committee with the ethical approval code of “IR.RAZI.REC.1401.014” and conducted from May 2022 to September 2022 in Kermanshah (Iran) following the Declaration of Helsinki. All the participants signed the written informed consent form, and the trial was registered with the registration code “IRCT20210617051606N7”.

**Table 1:** The characteristics of the participants

Variables	Experimental Groups			Statistics
	Anodal + AE (*n* = 12)	Sham + AE (*n* = 12)	Control (*n* = 12)	
Age _(years)_	27.75 ± 4.9	26.33 ± 7.1	25.25 ± 7.2	F(2) = 0.45, *p* = 0.64
Body Mass _(kg)_	78.33 ± 4.9	74.50 ± 7.0	75.00 ± 6.9	F(2) = 1.27, *p* = 0.29
Height _(cm)_	166.33 ± 5.0	165.25 ± 4.1	166.41 ± 7.2	F(2) = 0.16, *p* = 0.85
BMI _(kg/m_^2^_)_	28.31 ± 1.3	27.24 ± 1.8	27.07 ± 1.8	F(2) = 1.80, *p* = 0.18
TCS _(Score)_	112.75 ± 3.6	113.25 ± 3.2	112.91 ± 4.5	F(2) = 1.87, *p* = 0.17

Note: the data is reported as mean ± standard deviation. **AE**: Aerobic Exercise, **BMI**: Body Mass Index, **TCS**: Total Craving Score

### Randomization, allocation, and concealment

In this study, permuted block randomization was conducted using the website www.randomization.com. Each participant was first assigned a unique identifier code, and a 36-digit sequence (matching the total sample size) was generated. Treatment labels, including [1] Anodal stimulation + exercise [2], Sham stimulation + exercise, and [3] Control, were entered into the appropriate section of the website. To mitigate potential issues related to equal block sizes, permuted block randomization with varying block sizes was employed. The block sizes were unequal and represented multiples of the number of treatment groups (e.g., 2, 4, or 6). The website randomly generates sequences of blocks with varying sizes. Upon executing the “Generate Plan” function, participants were randomly allocated to blocks of differing sizes, each containing a randomized treatment sequence. Finally, treatment assignments were determined by matching participant ID codes with the generated blocks (12 participants per group).

Blinding was performed by having different researchers apply tDCS (the only one who knew the tDCS condition) and assess the outcome measures. To do that, when the tDCS intervention was about to start, the outcome assessor would leave the room and not come back until the respective tDCS condition was finished, the tDCS device was turned off, and the electrodes had been removed from participants’ heads. No information exchange was performed between them. Moreover, participants were not told of which experimental condition they were receiving and the tDCS device was kept behind them (out of sight) and covered so that participants could not see the device and any information on its display. To evaluate the effectiveness of the blinding, at the end of the 5th stimulation session, the participants completed a questionnaire provided by Fertonani et al. [45] regarding the sensations and intensity levels experienced during stimulation. Analysis of the results of the questionnaire showed that there were no serious side effects or adverse effects. Moreover, there were no significant differences in tDCS-induced sensations and active guessing rate between anodal and sham tDCS, indicating the effectiveness of the blinding strategy. It’s worth noting that we have used this blinding strategy in our previous studies, and its efficacy has been demonstrated in those studies as well [46–49].

### Transcranial direct current stimulation (tDCS)

**Fig. 3:**
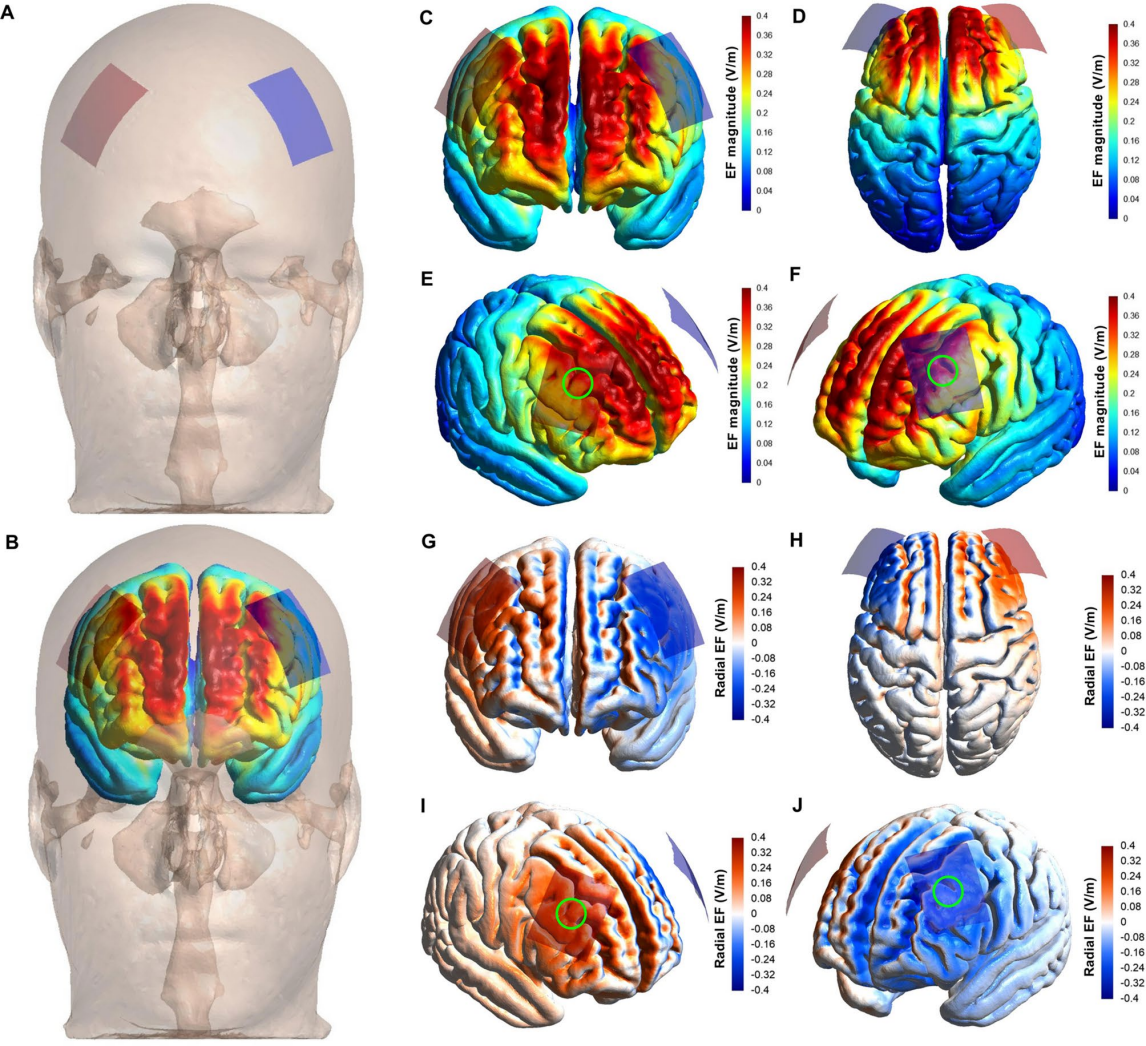
The electric field magnitude and radial component induced by the tDCS montage. Analysis of tDCS-induced strength and radial (normal to the cortical surface) component of the electric field (EF) using a head model (MNI152) developed from magnetic resonance imaging. Electrode montage targeting anodal tDCS in the right dorsolateral prefrontal cortex. Anodal (red rectangle; 5 × 4 cm) and cathodal (blue rectangle; 5 × 4 cm) electrodes were placed over the scalp (**A** and **B**). The magnitude of the EF is shown in panels C-F, with hot colors (e.g., red) representing stronger EF and cold colors (e.g., blue) representing weaker EF. Panels G-J depict the radial EF, with red representing the electric current flowing into the cortex (i.e., inducing excitatory effects) and blue representing the electric current flowing out of the cortex (i.e., inducing inhibitory effects). Panels E and F show that the research montage reached the target area with enough electric current magnitude to generate a neuromodulatory effect (green circles roughly indicating the target locations). Furthermore, as shown in panels I and J, the target areas were stimulated with the desired polarity (i.e., anodal current) to elicit excitatory effects in the target regions (green circles roughly indicating the target areas)

The “Anodal + AE” and “Sham + AE” groups received five consecutive sessions of anodal or sham tDCS, respectively. Both “participants” and “outcome assessors” were blinded to the type of stimulation. A battery-driven stimulator (NeuroStim 2, Medina Tebgostar, Tehran, Iran) delivered 2 mA of tDCS for 20 min. Two 4 × 5 cm carbon electrodes (0.1 mA/cm²) covered with saline-soaked sponges (NaCl 140 mmol in Milli-Q water) were used as an anode and cathode [46–48]. As with research advocating its effectiveness in reducing food and drug cravings [27, 36, 50], the + F4/-F3 montage was applied by placing the anode over F4 (right DLPFC) and the cathode over F3 (left DLPFC; Figs. 3A-B). The brain current flow during tDCS was modeled using SimNIBS 4.0.0 [51] following the procedures reported in other research [15, 46–49, 52], confirming that the right DLPFC receives adequate electric field intensity (Figs. 3C-F) and inward current (Figs. 3G-J) to induce neuromodulation [53, 54]. Other prefrontal regions (i.e., the bilateral ventromedial and ventrolateral PFC and left DLPFC) were similarly affected (Fig. 3). A 64-channel EEG cap (10–20 system) was located at the target regions. In anodal stimulation, the current ramped up over 30 s, held at 2 mA for 20 min, and then ramped down over 30 s. In the sham condition, the current was active for only 30 s before ramping down. Notably, this sham procedure effectively blinded participants [46, 55–57].

### Aerobic exercise (AE)

Following five days of tDCS, participants in the Anodal + AE and Sham + AE groups undertook a four-week supervised aerobic exercise program consisting of continuous jogging, performed three times per week on alternate days [58]. Exercise intensity was monitored using both heart rate reserve and ratings of perceived exertion (RPE) on the Borg scale [6–20]. Participants were instructed to use the carotid pulse method and report their RPEs. Resting heart rate was measured across three days, and maximum heart rate was estimated using the formula 220 minus age. The target heart rate was calculated using the Karvonen formula: ***Target Heart Rate = [(Maximum Heart Rate – Resting Heart Rate) × %Intensity] + Resting Heart Rate***. This method has been shown to effectively control exercise intensity in previous studies [59]. Before beginning the aerobic program, participants completed a familiarization session in which they were trained on the session structure, including how to report RPE and jog at the prescribed intensity based on their heart rate and perceived exertion. Each session consisted of three phases: a warm-up (7–10 min of slow jogging and stretching), the main exercise phase (jogging at individualized intensity), and a cool-down (five minutes of slow jogging and static stretching). During the main exercise, participants paused every three minutes to measure their carotid pulse for 15 s and then resumed jogging. The clinical exercise physiologist supervising the sessions adjusted participants’ jogging pace if their heart rate deviated by ± 5 beats from the target. The aerobic exercise sessions were conducted outdoors in a quiet setting. Detailed parameters of the exercise protocol, including frequency, intensity, time, and type, are presented in Table 2. All sessions were supervised by a clinical exercise physiologist.

**Table 2:** Aerobic exercise characteristics including frequency, intensity, time, and type of exercise

		Exercise Parameters (FITT)			
Time	Frequency (Per Week)		Intensity (HHR and 6–20 RPE)	Time (Min)	Type
Week 1	3		50–60% of HRR; RPE: 12–13	20	Jogging
Week 2	3		50–60% of HRR; RPE: 12–13	25	Jogging
Week 3	3		50–60% of HRR; RPE: 12–13	30	Jogging
Week 4	3		50–60% of HRR; RPE: 12–13	35	Jogging

**HRR**: Heart Rate Reserve, **RPE**: Rating of Perceived Exertion

### Food craving

Both instruments used to assess food cravings, the 15-item Food Craving State Questionnaire and the 12-item Craving Visual Analog Scale Color Picture Questionnaire, have demonstrated strong psychometric properties and are widely utilized in both clinical and experimental settings for evaluating momentary food cravings [43, 60]. The Food Craving State Questionnaire consists of 15 items assessing the intensity of current food craving, with Likert-type responses ranging from 1 to 5, resulting in total scores between 15 and 75 [36, 61]. In the Craving Visual Analog Scale, participants viewed 12 images from the International Affective Picture System and rated how much they “would like to eat each food right now” on a 5-point scale, yielding total scores between 12 and 60. The images were equally distributed among carbohydrates, fast foods, high-fat foods, and sweets [36]. Visual processing of food has been shown to activate the same brain networks involved in tasting and smelling, supporting the use of visual stimuli for measuring cravings [62, 63]. The questionnaires were completed two hours before the participants’ main meal (i.e., lunch) under standardized and consistent conditions to control for potential confounding factors. Higher scores on both scales reflected stronger cravings. The combined total score from the two food craving questionnaires was used as an indicator of food craving symptoms and served as one of the inclusion criteria for this study.

### Impulsivity toward non-food images

The computerized Go/No-Go task (Medina Tebgostar, Tehran, Iran), a validated and widely used tool for assessing response inhibition or inhibitory control in cognitive and behavioral neuroscience research [64, 65], was employed to measure impulsivity toward non-food images [15]. Participants were seated approximately 80 cm from the screen, with adjustments made to ensure optimal viewing conditions. The task consisted of 100 trials in which the letter “P” (Go) or “R” (No-Go) was presented on the screen for one second. Letters were displayed in black at the center of a colored square, green for “P” and yellow for “R”. Each square measured 4 cm × 4 cm and appeared at the center of the screen. Participants were instructed to click on the square when the letter “P” appeared and to withhold their response when “R” appeared. As “P” occurred in the majority of trials, the task evaluated participants’ ability to inhibit automatic responses [64]. The task lasted approximately 15 min and was conducted under strictly controlled laboratory conditions, free from distractions. Higher impulsivity scores reflected better inhibitory control and lower impulsivity.

### Risky decision-making

The Persian version of the computerized Iowa Gambling Task (Medina Tebgostar, Tehran, Iran) was used to assess risky decision-making [15]. The Iowa Gambling Task is a well-validated neuropsychological instrument originally developed to evaluate decision-making impairments in patients with ventromedial prefrontal cortex damage, and it has since been widely employed to assess decision-making and risk-taking behaviors in both clinical and non-clinical populations [66–70]. For the task, participants sat comfortably approximately 80 cm from the screen, with adjustments made to ensure optimal viewing conditions. The test included 100 trials, an initial virtual loan of $2,000, and an intertrial interval of approximately 2.5 s for reward-only trials or 5 s when a reward was followed by a penalty. In each trial, participants selected a card from one of four decks, Deck A, B, C, or D, each associated with different probabilities of winning and losing. Participants were unaware of the specific outcomes linked to each deck. They were instructed that their goal was to maximize profit beyond the initial loan, without knowing in advance when the test would end. After each selection, a message displayed the amount of money won; if the selection involved a loss, a separate message appeared a few seconds later indicating the amount lost. The task lasted approximately 15 min and was administered under controlled laboratory conditions free of external distractions. Higher total scores indicated better decision-making performance [68].

### Cognitive flexibility

The Cognitive Flexibility Inventory (CFI), a well-validated 20-item self-report questionnaire with strong internal consistency, convergent validity, and test-retest reliability, was used to assess cognitive flexibility [71]. The CFI evaluates three core aspects of cognitive flexibility: [1] perceiving difficult situations as controllable [2], recognizing multiple explanations for life events and behaviors, and [3] identifying various solutions to challenges. Each item is rated on a 7-point Likert scale (1 to 7), yielding total scores ranging from 20 to 140. Participants received clear instructions on how to complete the inventory. The task took approximately five minutes to complete and was administered under controlled laboratory conditions, free from external distractions. Higher scores indicate greater cognitive flexibility.

### Statistical analysis

Data are presented as mean ± standard deviation (M ± SD). Normal distribution was assessed using the Shapiro-Wilk test, and all data followed a normal distribution. A two-way mixed ANOVA (3 × 4 factorial design; 3 groups, 4 time points) was used to analyze food cravings and cognitive flexibility, and a 3 × 3 design was used for impulsivity and risky decision-making. When a significant group × time interaction was found, Bonferroni post hoc tests were applied for pairwise comparisons. The Greenhouse-Geisser correction was used when the sphericity assumption was violated. Partial eta squared (ɳ²p) measured effect size and was interpreted as small (0.01–0.059), medium (0.06–0.139), or large (≥ 0.14). Cohen’s d was used for pairwise comparisons and categorized as small (0.20–0.49), medium (0.50–0.79), or large (≥ 0.80) [72]. Statistical analyses were performed with SPSS 27, with p˂0.05 considered significant.

## Results

The overall results for the study variables are presented in Table 3, and detailed ANOVA results are given in the Supplementary Table. All participants completed the whole experimental procedure with no dropouts.

**Table 3:** Outcome variables at specified time points in three different groups (*n* = 12 per group, 36 in total)

Variables| Time	Groups	Baseline (Day 0)	Post tDCS (Day 6)	Post exercise (Day 38)	Follow-up (Day 68)
Food Craving Questionnaire State _(Score)_	a-tDCS + AE	59.5 ± 6.2	53.5 ± 5.9*^,&^	42.8 ± 5.3*^,&^	52.3 ± 5.0*^,&^
	Sham + AE	61.8 ± 3.5	60.83 ± 4.5	49.7 ± 3.9*	60.0 ± 4.0
	Control	63.3 ± 4.5	63.2 ± 3.9	62.3 ± 4.9	63.8 ± 4.5
Craving Visual Analog Scale _(Score)_	a-tDCS + AE	53.3 ± 5.7	47.9 ± 4.9*	39.4 ±4.1*^,&^	45.4 ± 5.1*
	Sham + AE	51.4 ± 3.9	50.66 ± 3.7	44.1 ± 4.6*	49.8 ± 4.3
	Control	52.3 ± 2.8	52.4 ± 3.5	51.3 ± 3.4	50.4 ± 2.9
Cognitive Flexibility _(Score)_	a-tDCS + AE	99.8 ± 13.5	112.8 ± 11.9	132.8 ± 9.4*^,&^	120.0 ± 11.2*
	Sham + AE	102.4 ± 13.3	104.8 ± 13.3	118.4 ± 10.0*	109.3 ± 10.4
	Control	104.5 ± 25.0	103.9 ± 20.8	104.4 ± 17.4	102.4 ± 17.3
Impulsivity _(Score)_	a-tDCS + AE	35.6 ± 2.8	39.5 ± 2.6*^,&^	46.4 ± 2.2*^,&^	-
	Sham + AE	33.8 ± 5.1	36.1 ± 3.8	41.1 ± 3.3*	-
	Control	34.9 ± 2.1	35.8 ± 2.7	34.4 ± 2.2	-
Iowa Gambling Test _(Score)_	a-tDCS + AE	1342.9± 533.9	1902.9± 652.3	2258.3 ±875.1*	-
	Sham + AE	1301.6 ± 783.1	1362.5 ± 711.0	1661.2 ± 729.6	-
	Control	1392.5 ± 540.0	1444.1 ± 421.1	1415.4 ± 415.3	-
Body Mass _(kg)_	a-tDCS + AE	78.3 ± 4.9	-	74.5 ± 5.4	-
	Sham + AE	74.5 ± 7.0	-	72.3 ± 6.7	-
	Control	75.0 ± 6.9	-	75.4 ± 6.9	-

Note: the data is reported as mean ± standard deviation. **AE**: Aerobic Exercise; **a-tDCS**: anodal Transcranial Direct Current Stimulation; **⁕** = significantly different from the control group (p˂0.05); **&** = significantly different from the sham + AE group (p˂0.05)

### Food craving symptoms

**Fig. 4:**
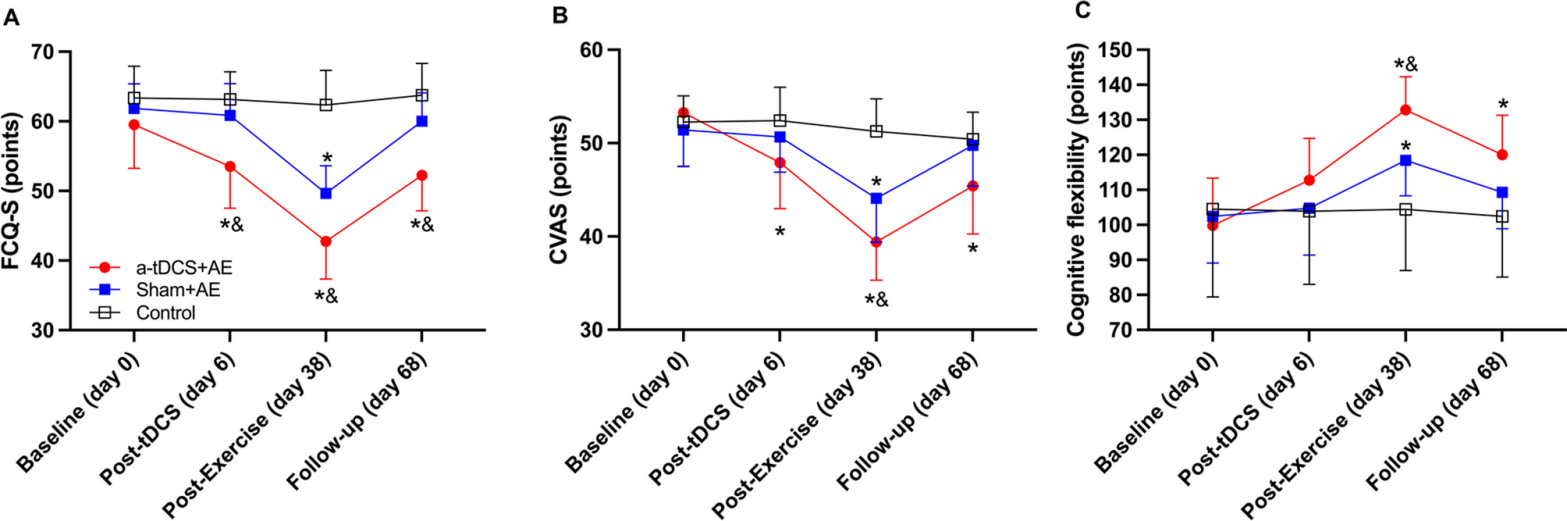
Food Craving Questionnaire State **(A)**, Craving Visual Analog Scale **(B)** and cognitive flexibility **(C)** at baseline, post Transcranial Direct Current Stimulation (tDCS), post aerobic exercise (AE) training, and one-month follow-up. ∗ = significantly different from the control group (p˂0.05); & = significantly different from the sham + AE group (p˂0.05)

A significant main effect of group, time, and interaction was observed on the food craving (15-item food craving state questionnaire) (all p˂0.001; Fig. 4A). After 5 days of tDCS intervention, the food craving in the Anodal + AE group was significantly lower than in the Sham + AE (*p* = 0.003, d = 1.4) and control groups (p˂0.001, d = 1.9). Following 4 weeks of aerobic exercise, both Anodal + AE and Sham + AE groups showed significantly lower food craving compared to the control (p˂0.001, d = 3.8 and p˂0.001, d = 2.8, respectively), with Anodal + AE also being lower than Sham + AE (*p* = 0.004, d = 1.5). One-month post-AE program, the food craving in the Anodal + AE remained significantly lower than in Sham + AE (p˂0.001, d = 1.7) and control (p˂0.001, d = 2.4), while no significant difference was found between Sham + AE and control groups (*p* = 0.16).

A significant main effect of group, time, and interaction was found on the food craving (12-item Craving Visual Analog Scale) (all *p* ≤ 0.01; Fig. 4B). Pairwise comparisons showed that 5 days post-tDCS intervention, the food craving was significantly lower in the Anodal + AE group compared to the control (*p* = 0.035, d = 1.0). After 4 weeks of AE, both Anodal + AE and Sham + AE groups had significantly lower food craving compared to control (p˂0.001, d = 3.1 and p˂0.001, d = 1.8, respectively), with Anodal + AE also lower than Sham + AE (*p* = 0.027, d = 1). One-month post-AE, the food craving remained significantly lower in the Anodal + AE group compared to the control (*p* = 0.02, d = 1.25).

### Cognitive flexibility

Significant main effects of time and interaction were observed on cognitive flexibility (all p˂0.005; Fig. 4C), with no main effect of group (*p* = 0.11). Pairwise comparisons showed that after 4 weeks of aerobic exercise, cognitive flexibility was significantly higher in both Anodal + AE (p˂0.001, d = 2.1) and Sham + AE (*p* = 0.035, d = 1.0) groups than in control, and higher in Anodal + AE compared to Sham + AE (*p* = 0.029, d = 1.4). One-month post-AE, cognitive flexibility remained significantly higher in Anodal + AE than in control (*p* = 0.009, d = 1.2).

### Impulsivity toward non-food images

**Fig. 5:**
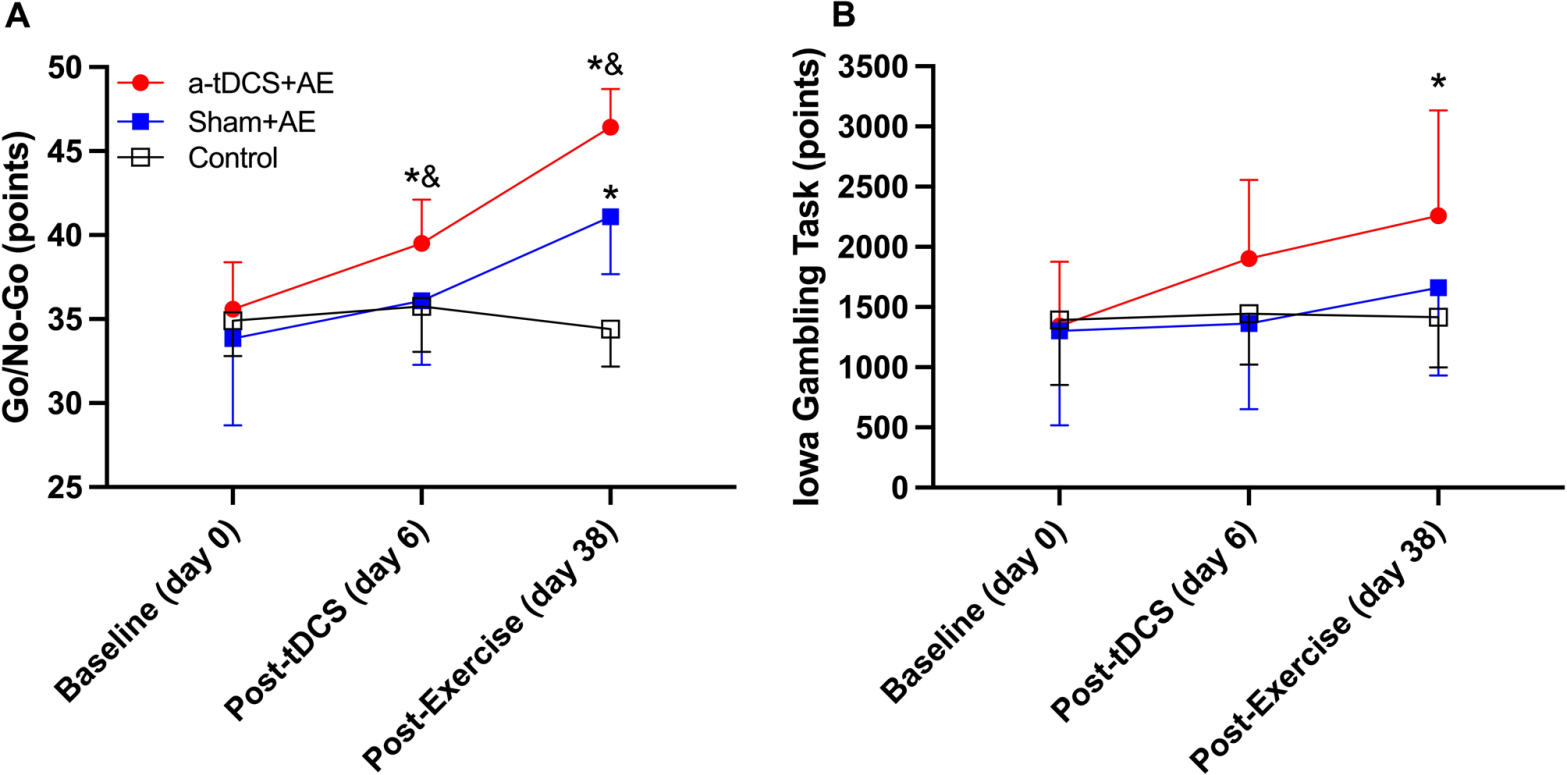
Impulsivity **(A)** and Risky Decision-Making **(B)** at baseline, post Transcranial Direct Current Stimulation (tDCS), and post aerobic exercise (AE) training. ∗ = significantly different from the control group (p˂0.05); & = significantly different from the sham + AE group (p˂0.05)

Significant main effects of group, time, and interaction were observed on impulsivity toward non-food images (all p˂0.001; Fig. 5A). Pairwise comparisons showed that, 5 days post-tDCS intervention, impulsivity toward non-food images was significantly higher in the Anodal + AE group compared to Sham + AE (*p* = 0.032, d = 1) and control groups (*p* = 0.016, d = 1.4). After 4 weeks of aerobic exercise, impulsivity toward non-food images was significantly higher in both Anodal + AE and Sham + AE groups than in control (p˂0.001, d = 5.4 and p˂0.001, d = 2.4, respectively), with Anodal + AE also higher than Sham + AE (p˂0.001, d = 1.9).

### Risky decision-making

Significant main effects of time and group × time interaction was found on risky decision-making (all *p* < 0.005; Fig. 5B), with no main effect of group (*p* = 0.14). Pairwise comparisons indicated that, after 4 weeks of aerobic exercise, risky decision-making was significantly higher in the Anodal + AE group compared to the control (*p* = 0.017, d = 1.3).

## Discussion

This study revealed the synergistic effects of multisession anodal tDCS followed by four weeks of aerobic exercise on food cravings, impulsivity toward non-food images, risky decision-making, and cognitive flexibility in women with overweight and obesity exhibiting food craving symptoms. Notably, these synergistic effects persisted for one month after the interventions concluded.

Our results demonstrated significantly lower food craving symptoms in the Anodal + AE group compared to the control group following five consecutive sessions of anodal tDCS. Evidence supports the efficacy of anodal tDCS in reducing food craving, food preference, and appetite control [9, 27, 73], which has been linked to enhanced cognitive control [27], increased dopaminergic pathway activity [36, 74], changes in pyramidal cell membrane potential affecting GABA release [75], and reductions in Na, Cl, K, and Ca surface masses in targeted brain regions [76], all contributing to neuroplasticity [77]. Notably, recent studies have reported sustained effects following consecutive (3 to 15) sessions of tDCS [36–38, 78]. For example, Ljubisavljevic et al. [36] reported reductions in food cravings lasting for one month after five consecutive anodal tDCS sessions over the right DLPFC, presumably due to tDCS-induced regulation of brain-derived neurotrophic factor. Multisession tDCS is further believed to enhance information processing efficiency within neural circuits, promoting protein synthesis and long-term potentiation [77]. Accordingly, the five-session tDCS protocol used in the present study may have induced favorable neural adaptations that contributed to reduced food craving symptoms. Additionally, this protocol may have preconditioned the brain for sustained synergistic effects when combined with four weeks of aerobic exercise, as evidenced by significantly lower food cravings in the Anodal + AE group compared to both the Sham + AE and control groups at 48 h and one month post-intervention. Nonetheless, it is important to acknowledge that while food cravings significantly declined, actual food intake was not directly measured in this study. Therefore, it remains uncertain whether such reductions in cravings translate into meaningful behavioral changes in dietary consumption.

There has been growing interest in multidimensional approaches that integrate various intervention strategies for the treatment of clinical conditions [39]. Such approaches can elicit synergistic effects, thereby enhancing health benefits [59, 79, 80]. For these benefits to be considered synergistic, however, the interventions must share common mechanistic pathways [39]. Notably, both tDCS and aerobic exercise have been shown to synergistically promote cortical neuroplasticity, a key factor in the brain’s adaptive responses to novel stimuli [39, 40]. For example, Ward et al. [81] reported that the combined application of chronic tDCS and aerobic exercise significantly enhanced several cognitive functions in healthy young adults. Moreover, combining tDCS with aerobic exercise has demonstrated greater efficacy than either intervention alone in various domains, including reduced pain perception [82], diminished acute appetite sensation [83], enhanced executive function [39], delayed cognitive decline and dementia [41], and improved information processing speed and executive control [84].

In this study, an anodal tDCS montage of + F4/−F3 was applied over five consecutive sessions. This montage aligns with the “right brain hypothesis of obesity,” which posits that increased activity in the right DLPFC enhances inhibitory control, thereby reducing food craving and suppressing overeating [36]. It has been shown to effectively modulate targeted neural circuits and facilitate long-term potentiation, a key mechanism underlying neuroplasticity [85]. In contrast, the aerobic exercise program likely activates broader brain networks through reticular arousal pathways and neural oscillatory shifts [39]. The convergence of these pathways may enhance executive functions and inhibitory control, potentially explaining the observed synergistic effects of tDCS and aerobic exercise compared to aerobic exercise alone or control [39, 40]. This hypothesis is supported by our findings related to impulsivity, risky decision-making, and cognitive flexibility.

Our results indicated reduced impulsive behavior toward non-food images in the Anodal + AE group compared to both Sham + AE and control groups, as assessed after five sessions of brain stimulation and again 48 h post-exercise. Impulsivity is associated with hypoactivation of the DLPFC and diminished inhibitory control, both of which are critical factors in food craving and overeating, particularly among individuals with overweight and obesity [86]. Recent systematic reviews have highlighted the promising effects of neuromodulation in addressing impulsivity, binge eating, and impaired response inhibition [87, 88]. Supporting this, fMRI studies have shown that anodal tDCS over the right DLPFC enhances response inhibition by modulating neural circuit activity and improving connectivity in the frontal-basal ganglia network, the right DLPFC, and the right inferior parietal cortex [89]. Additionally, tDCS can enhance cognitive function by influencing the resting membrane potential [90]. Physical activity also improves executive functions and inhibitory control in both healthy and clinical populations [91–93]. Specifically, aerobic exercise enhances cognitive function by increasing catecholamine release and activating broader neural networks related to inhibitory control, some of which may not be accessible through tDCS alone [39]. Our findings support this, as the Sham + AE group exhibited lower impulsivity throughout the intervention. The results further emphasized the synergistic effects of combining tDCS and aerobic exercise, as evidenced by significantly lower impulsivity in the Anodal + AE group compared to the Sham + AE group after the intervention. Previous studies have reported sustained effects of repeated tDCS sessions on memory, working memory, and food craving lasting one to three months [36–38]. These sessions are believed to generate cumulative effects that initiate long-term potentiation and neuroplasticity in targeted brain regions. The tDCS protocol used in this study, five consecutive sessions of + F4/−F3 anodal tDCS, likely induced such effects in areas responsible for inhibitory control, potentially preconditioning the brain to respond more effectively to the subsequent four weeks of aerobic exercise through the convergence of mechanistic pathways from both interventions.

The results indicated that risky decision-making was significantly improved in the Anodal + AE group compared to the control group at the end of the intervention. Decision-making is a complex, multidimensional process requiring coordinated activation of various brain regions, including the DLPFC, medial prefrontal cortex, and orbitofrontal cortex [94]. Among these, the DLPFC plays a central role in regulating risky decision-making [15]. Poor decision-making has been associated with food craving, overeating, and obesity, collectively contributing to a risk model for obesity [95–98]. A recent systematic review and meta-analysis reported that neuromodulation of the DLPFC positively influences risk-taking behavior, resulting in more conservative, risk-averse responses [99]. For example, a single session of anodal tDCS over the DLPFC improved risky decision-making in female sports referees [15], and six sessions of + F4/−F3 tDCS enhanced decision-making in patients with gambling disorders [33]. This positive influence on risky decision-making is thought to be mediated by increased activity in the right DLPFC and enhanced dopamine release in the right ventral striatum [99]. Regular aerobic exercise has also been shown to positively influence decision-making, likely through the improvement of executive functions essential for making decisions under conditions of ambiguity and risk [100, 101]. Notably, significant improvements in risky decision-making were observed only when tDCS and aerobic exercise were combined, underscoring the potential synergistic effects of these interventions. We propose that the tDCS protocol used in this study served as a preconditioning stimulus for executive functions, leading to the observed improvements in risky decision-making in the Anodal + AE group compared to the control condition.

Our results indicated that cognitive flexibility was not significantly improved following tDCS alone. Prior studies have reported enhancements in decision-making, cognitive flexibility, and inhibitory control after tDCS over the DLPFC [31, 32]. This discrepancy may be attributed to differences in assessment methods; while previous studies employed neuropsychological tasks, we used self-report measures via a cognitive flexibility questionnaire. Hohl et al. [102] noted a limited correlation between neuropsychological task performance and self-reported cognitive flexibility, suggesting these tools may capture different aspects of the construct. Another possible explanation involves the timing of assessment; our study measured cognitive flexibility 24 h after the fifth tDCS session, whereas earlier studies conducted assessments immediately post-stimulation. In contrast, the observed improvement in cognitive flexibility after exercise training, particularly when combined with tDCS, and the sustained enhancement in the Anodal + AE group at the one-month follow-up highlight the potential synergistic effect between tDCS and aerobic exercise, representing one of the novel findings of this study. Cognitive flexibility refers to the ability to rapidly adjust cognitive processes to optimize executive functioning in response to changing task demands [96]. It encompasses both proactive and reactive control mechanisms, which are essential for regulating food craving and appetite [17, 103]. Impairments in cognitive flexibility have been associated with overweight and obesity, resulting in diminished control over food craving and appetite, and ultimately contributing to weight gain [17, 104]. A substantial body of research has documented the positive effects of aerobic exercise on cognitive flexibility and executive functions across both healthy and clinical populations [25, 101, 105–107]. These improvements may be mediated by several mechanisms, including increased synthesis and secretion of brain-derived neurotrophic factor, enhanced cortical excitability, improved neuroplasticity, structural brain adaptations, elevated psychosocial functioning, and antidepressant effects [25, 101, 108, 109]. Neuroimaging studies have underscored the DLPFC’s critical role in cognitive flexibility, executive functioning, and cognitive control, making it a compelling target for neuromodulation strategies [110]. It appears that the aerobic exercise protocol employed in this study activated some of these mechanisms, potentially amplified by the preceding five sessions of tDCS. Agboada et al. [85] discussed the emergence of early and late long-term potentiation (LTP) following acute and chronic brain stimulation, respectively. According to this model, a single tDCS session may induce early-phase LTP, while repeated sessions can lead to late-phase LTP. Thus, the five consecutive sessions of anodal tDCS likely induced favorable neuroplastic changes in the targeted brain regions, which may have synergized with the subsequent four weeks of aerobic exercise. Importantly, these effects persisted one month after the intervention.

All necessary precautions were taken to ensure rigorous control of study procedures. However, these findings should be interpreted with caution due to several limitations. First, the study included a small sample of women with overweight and obesity exhibiting food craving symptoms, limiting the generalizability of the results to other populations. Additionally, the inhibitory control tasks used were non-specific (e.g., not tailored to cue-reactivity), and no clinical diagnosis of food craving was conducted by a psychiatrist. Moreover, although reductions in food cravings were observed, actual food intake was not measured, preventing us from determining whether the intervention led to meaningful changes in dietary behavior. Another limitation is the absence of neurophysiological assessments, such as EEG, fNIRS, or fMRI, or measures of corticocortical, corticospinal, or motor neuronal excitability, which could have provided mechanistic insights into the effects of multisession tDCS and aerobic exercise. Furthermore, the study did not include a group receiving anodal tDCS alone, which could have clarified the specific contribution of tDCS independent of exercise. Future studies should address these limitations by incorporating more diverse samples, longer intervention durations, and objective measures of brain activity and eating behavior to better understand long-term adherence and outcomes. Despite these limitations, a key strength of this study lies in the use of a multisession tDCS protocol, whereas most prior research has focused on single-session applications. Importantly, the timing of tDCS administration, delivered prior to, rather than during, the aerobic training period, offers a novel and practical approach with potential for clinical translation.

The findings of this study highlight the potential clinical utility of combining multisession anodal tDCS with aerobic exercise as a novel intervention for reducing food craving and enhancing related cognitive and behavioral outcomes in individuals with overweight or obesity and symptoms of food craving. The observed synergistic effects suggest that this combined approach may offer greater and more sustained benefits than aerobic exercise alone, positioning it as a promising strategy for managing food cravings, impulsivity toward non-food stimuli, risky decision-making, and cognitive flexibility. Importantly, aerobic exercise alone demonstrated significant benefits, particularly in reducing food cravings and improving cognitive flexibility, underscoring its value as an effective standalone intervention. These findings support the integration of neuromodulation and physical exercise into clinical practice, especially for individuals struggling with food craving and weight management. Future research should investigate the independent effects of tDCS to further elucidate its specific contribution to these outcomes.

## Conclusions

The findings of this study suggest that five consecutive sessions of anodal tDCS (+ F4/−F3 montage), combined with four weeks of moderate-intensity continuous aerobic exercise, can effectively reduce food cravings in women with overweight or obesity and symptoms of food craving. This reduction was accompanied by improvements in impulsivity toward non-food stimuli, risky decision-making, and cognitive flexibility, effects that persisted for one month following the intervention. These results may inform the development of effective intervention strategies for weight management in individuals with overweight or obesity and food craving symptoms, including caregivers. Further research is warranted to elucidate the underlying mechanisms driving these outcomes.

## Electronic supplementary material

Below is the link to the electronic supplementary material.

Supplementary Material 1

## Data Availability

The data generated and/or analyzed during the current study are available from the corresponding author on reasonable request.

## Abbreviations

AE: Aerobic Exercise
ACSM: American College of Sports Medicine
CF: Cognitive Flexibility
CVAS: Craving Visual Analog Scale
DLPFC: Dorsolateral Prefrontal Cortex
FC: Food Craving
FCQ-S: Food Craving Questionnaire State
HRR: Heart Rate Reserve
IMP: Impulsivity
IGT: Iowa Gambling Task
LTP: Long-Term Potentiation
RPE: Rating of Perceived Exertion
RHR: Resting Heart Rate
RDM: Risky Decision-Making
THR: Target Heart Rate
tDCS: Transcranial Direct Current Stimulation
WHO: World Health Organization
