# Supplementary material for: Multisession transcranial direct current stimulation and aerobic exercise synergistically improve food craving symptoms, impulsivity, and cognitive flexibility in women with overweight and obesity: a randomized controlled trial
Source: Int J Behav Nutr Phys Act. 2025 Jun 2;22:68. doi: 10.1186/s12966-025-01773-0 (PMC12131640; doi:10.1186/s12966-025-01773-0)
Supplement: Supplementary file 1 — Supplementary Material 1 [file 12966_2025_1773_MOESM1_ESM.docx]

**Supplementary Table 1**. Statistical values description of the two-way mixed ANOVA of the main outcomes.

| Variable | Main effect of group | Main effect of time | Interaction |
| --- | --- | --- | --- |
| FCQ-S | *F_(2,33)_ = 19.1, p ˂ 0.001, ɳ^2^_p_ = 0.54* | *F_(1.8,59.8)_ = 139.9, p ˂ 0.001, ɳ^2^_p_ = 0.81* | *F_(6,99)_ = 31.7, p ˂ 0.001, ɳ^2^_p_ = 0.66* |
| CVAS | *F_(2,33)_ = 5.1, p = 0.011, ɳ^2^_p_ = 0.24* | *F_(2.3,76.9)_ = 102.1, p ˂ 0.001, ɳ^2^_p_ = 0.76* | *F_(6,99)_ = 26.5, p ˂ 0.001, ɳ^2^_p_ = 0.62* |
| Impulsivity | *F_(2,33)_ = 10.7, p ˂ 0.001, ɳ^2^_p_ = 0.39* | *F_(1.5,49.9)_ = 164.4, p ˂ 0.001, ɳ^2^_p_ = 0.83* | *F_(4,66)_ = 57.2, p ˂ 0.001, ɳ^2^_p_ = 0.77* |
| Risky decision-making | *F_(2,33)_ = 2.1, p = 0.14, ɳ^2^_p_ = 0.11* | *F_(1.3,46.1)_ = 11.0, p = 0.001, ɳ^2^_p_ = 0.25* | *F_(4,66)_ = 4.3, p = 0.004, ɳ^2^_p_ = 0.21* |
| Cognitive flexibility | *F_(1.8,62.6)_ = 44.0, p ˂ 0.005, ɳ^2^_p_ = 0.57* | *F_(2,33)_ = 2.4, p = 0.11, ɳ^2^_p_ = 0.13* | *F_(6,99)_ = 15.5, p ˂ 0.005, ɳ^2^_p_ = 0.49* |

Note: FCQ-S = 15-item Food Craving Questionnaire; CVAS = 12-item Visual Analogue Food Craving Questionnaire.
